# Skin cancers associated with autoimmune conditions among elderly adults

**DOI:** 10.1038/sj.bjc.6605733

**Published:** 2010-06-15

**Authors:** E Lanoy, E A Engels

**Affiliations:** 1 INSERM, U943, Paris, F-75013 France; 2 UPMC Univ Paris 06, UMR S943, Paris, F-75013 France; 3 Division of Cancer Epidemiology and Genetics, National Cancer Institute, 6120 Executive Boulevard, Room 7076, Rockville, MD 20892, USA

**Keywords:** skin neoplasm, autoimmune conditions, aged

## Abstract

**Background:**

Immunosuppression is a risk factor for certain skin cancers. Autoimmune conditions can involve the skin, and may involve immunosuppressive therapies.

**Methods:**

We conducted a population-based case–control study among elderly US adults using Surveillance, Epidemiology, and End Results-Medicare-linked data of 44 613 skin cancer cases and 178 452 frequency-matched controls. Medicare claims identified autoimmune conditions. Adjusted odds ratios (ORs) compared prevalence in cases and controls.

**Results:**

The most frequent autoimmune condition was rheumatoid arthritis (2.29%), which was associated with slightly increased risk of Merkel cell carcinoma (*N*=1977; OR (95%CI): 1.39 (1.10–1.74)). Risk of cutaneous non-Hodgkin's lymphoma (*N*=2652) was increased with psoriasis (OR (95%CI): 3.20 (2.62–3.92)). Risk of Kaposi's sarcoma (*N*=773) was elevated with ulcerative colitis (OR (95%CI): 2.76 (1.42–5.39)), and risk of other sarcomas (*N*=1324) was elevated with Graves disease (2.62 (1.30–5.31)).

**Conclusions:**

These findings suggest that immune disturbances in the skin, arising from autoimmune conditions or their treatment, promote development of skin cancer.

Established risk factors for skin cancer include exposure to solar ultraviolet radiation, white race, and advancing age (Gruber and Armstrong). Immunosuppression also increases the risk of certain skin cancers. The risk in solid organ transplant recipients and people with human immunodeficiency virus (HIV) infection is extremely high for Kaposi's sarcoma (KS), a cutaneous tumour caused by human herpesvirus 8, somewhat elevated for cutaneous non-Hodgkin's lymphoma (NHL), Merkel cell carcinoma, and appendageal skin carcinomas and somewhat increased for melanoma (Lanoy *et al*, 2010).

Autoimmune conditions may also increase skin cancer risk. Chronic cutaneous inflammation that can characterise some autoimmune conditions (including psoriasis and scleroderma, which directly involve the skin) may plausibly cause DNA damage that could promote development of skin cancer. Immunosuppressive medications used to treat autoimmune conditions could have an additional role. This study aimed to investigate associations between autoimmune conditions and the subsequent risk of skin cancers among elderly US adults (aged 67 years and over).

## Materials and methods

We selected subjects from the Surveillance, Epidemiology, and End Results (SEER)-Medicare data set, which links SEER cancer registry and Medicare claims data (Warren *et al*, 2002). Skin cancer cases (other than basal and squamous cell carcinomas) and cancer-free controls were selected as described elsewhere (Lanoy *et al*, 2010), updated to include SEER Medicare data through 2005. The presence of autoimmune conditions before cancer diagnosis/control selections was assessed using Medicare claims data: rheumatoid arthritis (International Classification of Disease version 9 codes 714.0-714.3, 714.81, V82.1), Sjörgren's syndrome (710.2), systemic lupus erythematosus (710.0), polymyalgia rheumatica (725), giant cell arteritis (446.5), Addison's disease (255.4), Graves disease (242.0), psoriasis (696.0-696.1), localised scleroderma (701.0), Crohn's disease (555), ulcerative colitis (556), and pernicious anaemia (281.0).

We used polytomous logistic regression to derive odds ratios (ORs) comparing the prevalence of each medical condition in skin cancer cases to controls (Lanoy *et al*, 2010). We focused on associations that met statistical significance after Benjamini and Hochberg correction to account for multiple comparisons (*P*-value <0.05, after correction based on 6 skin cancers subtypes × 12 autoimmune conditions=72 tests) (Keselman *et al*, 2002), but we also present uncorrected 95%CIs for ORs that indicate associations of borderline significance (uncorrected *P*-value <0.05).

## Results

Characteristics of 44 613 skin cancer cases and 178 452 controls (corresponding to 134 779 unique control individuals) are shown in Table 1. Among 1540 appendageal carcinoma cases, the most frequent histological subtypes were sebaceous carcinoma (*N*=610), skin appendage carcinoma (*N*=316), and sweat gland adenocarcinoma (*N*=144). Of the 2652 cutaneous NHLs, 1854 were T-cell NHLs (of which 945 were mycosis fungoides/Sezary syndrome (MF/SS)) and 798 were B-cell NHLs. Among 1324 sarcomas, the most frequent histological subtypes were malignant fibrous histiocytoma (*N*=682), dermatofibrosarcoma (*N*=235), haemangiosarcoma (*N*=173), and leiomyosarcoma (*N*=121).

As shown in Table 2, an increased risk of Merkel cell carcinoma was observed in persons with rheumatoid arthritis (OR 1.39). Psoriasis was associated with an increased risk of cutaneous NHL (OR 3.20), caused by associations specifically with MF/SS (OR 5.81, 95% CI 4.43–7.63) and other cutaneous T-cell NHLs (OR 2.90, 95% CI 2.07–4.06), whereas cutaneous B-cell NHL risk was not elevated (OR 1.04, 95% CI 0.57–1.89). Ulcerative colitis was associated with risk of KS (OR 2.76), and an increased risk of sarcoma was found with Graves disease (OR 2.62). Among sarcomas, when we considered only malignant fibrous histiocytoma, the association with Graves disease remained significant (OR 3.24, 95% CI 1.33–7.89). Finally, melanoma risk was decreased in individuals with giant cell arteritis (OR 0.70). Table 2 also shows several additional associations of borderline significance.

## Discussion

We evaluated the risk of non-keratinocytic skin cancers in patients with autoimmune conditions in a case–control study among elderly US adults. Some associations that met our criterion for significance are likely to be explained by chronic inflammatory involvement of the skin or immune modulating therapies given for autoimmune conditions. The strong association that we observed between psoriasis and T-cell NHL (particularly MF/SS) is a biologically plausible manifestation of chronic inflammation (Ekstrom Smedby *et al*, 2008; Anderson *et al*, 2009). An alternative explanation could be misdiagnosis, in that cutaneous lymphoma can initially present as a chronic plaque-like lesion that might be mistaken for psoriasis (Ekstrom Smedby *et al*, 2008). In contrast, psoriasis was neither associated with cutaneous B-cell NHL nor, in a prior study (Anderson *et al*, 2009), with non-cutaneous T-cell NHL.

Our finding of elevated KS risk associated with ulcerative colitis is supported by several case reports in such patients receiving immunosuppressive drugs (Svrcek *et al*, 2009). The association of sarcoma with Graves disease mentioned above may relate to immunosuppressive therapies (Simon *et al*, 2009), as may that of rheumatoid arthritis with Merkel cell carcinoma, which is increased in HIV-infected people and transplant recipients (Lanoy *et al*, 2010) and may be caused by a recently discovered polyomavirus. Finally, we observed an unexpected significant deficit of melanoma among people with giant cell arteritis. A Danish study found no association between autoimmune diseases and melanoma incidence (Kaae *et al*, 2007), although others have reported an increased melanoma risk with pernicious anaemia (Brinton *et al*, 1989) and psoriasis (Stern, 2001).

The strength of our study is its large size, allowing us to evaluate associations between uncommon autoimmune conditions and skin cancers. Nonetheless, limitations of our study include that it was restricted to elderly adults, that basal and squamous cell skin cancers were not covered, and that we could not ascertain the presence of medical conditions below 65 years of age; we also had no data on immunosuppressive treatments.

Several associations identified between autoimmune conditions and skin cancer risk suggest that such conditions affecting the skin, or treated with immunosuppression promote the development of skin cancer; their further investigation will require additional large studies.

## Figures and Tables

**Table 1:** Characteristics of skin cancer cases and controls among elderly US adults

		**Skin cancer cases**					
	**Controls**	**Melanoma**	**Merkel cell carcinoma**	**Appendageal carcinomas**	**Cutaneous NHL**	**KS**	**Sarcomas**
	***N*=178 452**	***N*=36 092**	***N*=1977**	***N*=1540**	***N*=2652**	***N*=773**	***N*=1324**
*Gender, n (%)*							
Male	109316 (61.3)	22293 (61.8)	1186 (60.0)	785 (51.0)	1478 (55.7)	514 (66.5)	935 (70.6)
Female	69136 (38.7)	13799 (38.2)	791 (40.0)	755 (49.0)	1174 (44.3)	259 (33.5)	389 (29.4)
							
*Age in years, n (%)*							
67–69	28884 (16.2%)	6142 (17.0%)	182 (9.2%)	186 (12.1%)	443 (16.7%)	82 (10.6%)	158 (11.9%)
70–74	42792 (24.0%)	8957 (24.9%)	330 (16.7%)	311 (20.2%)	645 (24.3%)	136 (17.6%)	267 (20.2%)
75–79	43392 (24.3%)	8870 (24.6%)	439 (22.2%)	361 (23.4%)	661 (24.9%)	147 (19.0%)	309 (23.3%)
80–84	34928 (19.6%)	6838 (18.9%)	512 (25.9%)	317 (20.6%)	516 (19.5%)	171 (22.1%)	320 (24.2%)
85+	28456 (15.9%)	5285 (14.6%)	514 (26.0%)	365 (23.7%)	387 (14.6%)	237 (30.7%)	270 (20.4%)
Median age (years)	76	76	80	78	76	80	79
Calendar year of diagnosis/ selection, median (IQR)	2001 (1996–2003)	2001 (1996–2003)	2001 (1997–2003)	2000 (1993–2003)	2000 (1995–2003)	1998 (1993–2002)	2000 (1995–2003)
Duration of Medicare coverage, months, median (IQR)	95 (50–150)	93 (49–146)	118 (71–171)	106 (59–161)	93 (50–143)	90 (47–146)	103 (58–156)
Number of physician claims^a^, median (IQR)	37 (6–96)	38 (7–91)	65 (17–138)	51 (9.5–110.5)	40 (7–95)	33 (1–94)	43 (8–102)
Number of hospital claims^a^, median (IQR)	0 (0–2)	0 (0–2)	1 (0–3)	1 (0–2)	0 (0–2)	1 (0–2)	1 (0–2)
Number of outpatient claims^a^, median (IQR)	4 (0–14)	4 (0–13)	6 (1–20)	5 (0–16)	4 (0–14)	2 (0–10)	4 (0–15)

Abbreviations: IQR=interquartile range; KS=Kaposi's sarcoma; NHL=non-Hodgkin's lymphoma.

a The number of claims excludes the 12 months before skin cancer diagnosis (cases) or selection (controls).

**Table 2:** Associations of autoimmune conditions with skin cancer risk among elderly US adults

		**Associations with autoimmune condition: OR (95%CI) and number of subjects with the specified condition**					
	**Controls with condition (%)**	**Melanoma**	**Merkel cell carcinoma**	**Appendageal carcinomas**	**Cutaneous NHL**	**KS**	**Sarcomas**
	**N=178 452**	**N=36 092**	**N=1977**	**N=1540**	**N=2652**	**N=773**	**N=1324**
*Systemic/connective tissue*							
Rheumatoid arthritis	2.29	0.93 (0.86–1.01) *n*=758	1.39 (1.10–1.75)^a^ *n*=79	1.02 (0.74–1.39) *n*=42	1.15 (0.90–1.46) *n*=72	1.65 (1.09–2.49) *n*=25	1.13 (0.79–1.60) *n*=33
Sjörgren's syndrome	0.21	1.00 (0.77–1.30) *n*=73	1.46 (0.72–2.96) *n*<11	1.76 (0.83–3.74) *n*<11	1.02 (0.45–2.30) *n*<11	0.73 (0.10–5.22) *n*<11	1.97 (0.81–4.82) *n*<11
Systemic lupus erythematosus	0.20	0.80 (0.60–1.06) *n*=57	1.02 (0.42–2.47) *n*<11	1.62 (0.71–3.66) *n*<11	1.73 (0.91–3.27) *n*<11	1.65 (0.41–6.65) *n*<11	0.85 (0.21–3.45) *n*<11
Polymyalgia rheumatica	0.97	1.04 (0.92–1.17) *n*=353	1.03 (0.71–1.51) *n*=28	0.90 (0.55–1.46) *n*=17	0.71 (0.45–1.12) *n*=19	1.73 (0.99–3.01) *n*=13	0.74 (0.40–1.40) *n*<11
							
*Cardiovascular*							
Giant cell arteritis	0.30	0.70 (0.54–0.90)^a^ *n*=72	1.41 (0.79–2.50) *n*=12	0.83 (0.34–2.02) *n*<11	0.85 (0.40–1.80) *n*<11	0.87 (0.22–3.50) *n*<11	1.50 (0.67–3.38) *n*<11
							
*Endocrine*							
Addison's disease	0.13	1.06 (0.77–1.45) *n*=49	0.56 (0.14–2.27) *n*<11	1.28 (0.41–4.02) *n*<11	1.13 (0.42–3.05) *n*<11	1.98 (0.48–8.15) *n*<11	0.53 (0.07–3.83) *n*<11
Graves disease	0.24	0.88 (0.68–1.13) *n*=75	1.43 (0.73–2.78) *n*<11	0.67 (0.21–2.09) *n*<11	1.20 (0.59–2.43) *n*<11	0	2.62 (1.30–5.31)^a^ *n*<11
							
*Skin*							
Psoriasis	1.33	0.87 (0.78–0.97) *n*=418	1.29 (0.94–1.76) *n*=42	1.36 (0.94–1.97) *n*=30	3.20 (2.62–3.92)^a^ *n*=108	1.20 (0.66–2.21) *n*=11	0.99 (0.61–1.58) *n*=18
Localised scleroderma	0.14	0.85 (0.60–1.19) *n*=41	1.39 (0.57–3.40) *n*<11	1.11 (0.35–3.47) *n*<11	2.06 (1.01–4.19) *n*<11	2.34 (0.58–9.45) *n*<11	1.27 (0.31–5.15) *n*<11
							
*Gastrointestinal*							
Crohn's disease	0.27	1.12 (0.90–1.39) *n*<116	0.46 (0.15–1.45) *n*<11	2.25 (1.20–4.23) *n*<11	1.26 (0.65–2.46) *n*<11	1.09 (0.27–4.44) *n*<11	0.56 (0.14–2.26) *n*<11
Ulcerative colitis	0.48	1.08 (0.91–1.27) *n*=183	0.83 (0.44–1.56) *n*<11	0.98 (0.49–1.98) *n*<11	1.34 (0.82–2.17) *n*=17	2.76 (1.42–5.39)^a^ *n*<11	1.24 (0.61–2.49) *n*<11
Pernicious anaemia	1.57	0.90 (0.81–0.99) *n*=491	0.87 (0.63–1.20) *n*=39	0.84 (0.56–1.25) *n*=25	1.05 (0.78–1.43) *n*=44	0.77 (0.41–1.44) *n*<11	1.15 (0.78–1.71) *n*=26

Abbreviations: OR=odds ratio; CI=confidence interval; KS=Kaposi's sarcoma; NHL=non-Hodgkin's lymphoma.

Odds ratios are adjusted for age (67–69, 70–74, 75–79, 80–84, and 85–99 years), gender, selection year (1987–1993, 1994–1997, 1998–2000, and 2000–2002), and number of physician claims (0–4, 5–39, 40–109, and 110+). For consistency, all odds ratios are displayed to two decimal places, although in some instances the number of subjects with the specified medical condition is small. When the number of subjects with the autoimmune condition was between 1 and 10, the result is listed as ‘*n*<11’ to preserve subjects’ anonymity in accordance with the SEER-Medicare data use agreement.

a Association was significant after accounting for multiple testing using the Benjamini and Hochberg correction.

## References

[bib1] Anderson LA, Gadalla S, Morton LM, Landgren O, Pfeiffer R, Warren JL, Berndt SI, Ricker W, Parsons R, Engels EA (2009) Population-based study of autoimmune conditions and the risk of specific lymphoid malignancies. Int J Cancer 125: 398–405 19365835 10.1002/ijc.24287 PMC2692814

[bib2] Brinton LA, Gridley G, Hrubec Z, Hoover R, Fraumeni Jr JF (1989) Cancer risk following pernicious anaemia. Br J Cancer 59: 810–813 2736218 10.1038/bjc.1989.169 PMC2247229

[bib3] Ekstrom Smedby K, Vajdic CM, Falster M, Engels EA, Martinez-Maza O, Turner J, Hjalgrim H, Vineis P, Seniori Costantini A, Bracci PM, Holly EA, Willett E, Spinelli JJ, La Vecchia C, Zheng T, Becker N, De Sanjose S, Chiu BC, Dal Maso L, Cocco P, Maynadie M, Foretova L, Staines A, Brennan P, Davis S, Severson R, Cerhan JR, Breen EC, Birmann B, Grulich AE, Cozen W (2008) Autoimmune disorders and risk of non-Hodgkin lymphoma subtypes: a pooled analysis within the InterLymph Consortium. Blood 111: 4029–4038 18263783 10.1182/blood-2007-10-119974 PMC2288717

[bib4] Gruber SB, Armstrong BK (2006) Cutaneous, ocular melanoma. In Cancer Epidemiology, Prevention, Schottenfeld D, Fraumeni JF Jr E (eds) 3rd edn, pp 1196–1229. Oxford University Press: New York

[bib5] Kaae J, Wohlfahrt J, Boyd HA, Wulf HC, Biggar RJ, Melbye M (2007) The impact of autoimmune diseases on the incidence and prognosis of cutaneous malignant melanoma. Cancer Epidemiol Biomarkers Prev 16: 1840–1844 17855703 10.1158/1055-9965.EPI-07-0459

[bib6] Keselman HJ, Cribbie R, Holland B (2002) Controlling the rate of Type I error over a large set of statistical tests. Br J Math Stat Psychol 55: 27–39 12034010 10.1348/000711002159680

[bib7] Lanoy E, Costagliola D, Engels EA (2010) Skin cancers associated with HIV infection and solid organ transplant among elderly adults. Int J Cancer 126: 1724–1731 19810102 10.1002/ijc.24931 PMC2818159

[bib8] Simon Z, Ress Z, Toldi J, Trauninger A, Miltenyi Z, Illes A (2009) Rare association of Hodgkin lymphoma, Graves’ disease and myasthenia gravis complicated by post-radiation neurofibrosarcoma: coincidence or genetic susceptibility? Int J Hematol 89: 523–528 19381762 10.1007/s12185-009-0281-x

[bib9] Stern RS (2001) The risk of melanoma in association with long-term exposure to PUVA. J Am Acad Dermatol 44: 755–761 11312420 10.1067/mjd.2001.114576

[bib10] Svrcek M, Tiret E, Bennis M, Guyot P, Flejou JF (2009) KSHV/HHV8-associated intestinal Kaposi's sarcoma in patient with ulcerative colitis receiving immunosuppressive drugs: report of a case. Dis Colon Rectum 52: 154–158 19273971 10.1007/DCR.0b013e318197217f

[bib11] Warren JL, Klabunde CN, Schrag D, Bach PB, Riley GF (2002) Overview of the SEER-Medicare data: content, research applications, and generalizability to the United States elderly population. Med Care 40: IV–18 10.1097/01.MLR.0000020942.47004.03 12187163

